# Micro-compartmentalized strand displacement reactions with a random pool background

**DOI:** 10.1098/rsfs.2023.0011

**Published:** 2023-08-11

**Authors:** Thomas Mayer, Louis Givelet, Friedrich C. Simmel

**Affiliations:** Department of Bioscience, School of Natural Sciences, Technical University Munich, Garching, Germany

**Keywords:** DNA strand displacement, compartmentalization, chemical reaction networks

## Abstract

Toehold-mediated strand displacement (TMSD) is a widely used process in dynamic DNA nanotechnology, which has been applied for the actuation of molecular devices, in biosensor applications, and for DNA-based molecular computation. Similar processes also occur in a biological context, when RNA strands invade secondary structures or duplexes of other RNA or DNA molecules. Complex reaction environments—inside cells or synthetic cells—potentially contain a large number of competing nucleic acid molecules that transiently bind to the components of the strand displacement reaction of interest and thus slow down its kinetics. Here, we investigate the kinetics of TMSD reactions compartmentalized into water-in-oil emulsion droplets—in both the presence and absence of a random sequence background—using a droplet microfluidic ‘stopped flow’ set-up. The set-up enables one to determine the kinetics within thousands of droplets and easily vary experimental parameters such as the stoichiometry of the TMSD components. While the average kinetics in the droplets coincides precisely with the bulk behaviour, we observe considerable variability among the droplets. This variability is partially explained by the encapsulation procedure itself, but appears to be more pronounced in reactions involving a random pool background.

## 1. Introduction

Biochemical processes occurring in cells are subject to noise, which can arise from various sources such as stochastic gene expression [1,2], copy number variations [3] and other ‘extrinsic noise’ contributions. Noise has been observed in many biological contexts, and is thought to be used, for example, for decision making and bet-hedging [4,5]. In bottom-up biological systems and ‘synthetic cells’, noisy behaviour has been observed in the dynamics of compartmentalized biochemical reaction networks [6–8]. In synthetic cellular compartments with picolitre volumes or larger, however, molecule copy numbers are too high to result in an appreciable impact of stochastic reaction dynamics (caused by the stochasticity of the chemical reactions themselves), and noise has therefore been speculated to result, e.g. from the influence of the compartmentalization process on the activity of enzymes and variations in molecule number distributions [7,9].

**Figure 1.**
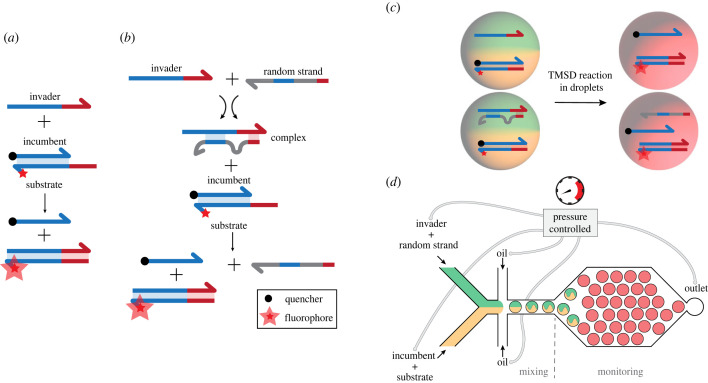
Toehold-mediated strand displacement with and without random pool strands. (*a*) Simplified depiction of a one-step TMSD process. The system consists of a single-stranded (ss) invader and a double-stranded (ds) reporter complex with a substrate strand which is labelled with a fluorophore and an incumbent labelled with a quencher. The short single-stranded overhang of the substrate, termed ‘toehold’ (depicted in red), is complementary to the toehold of the invader strand. Because of the sequence complementarity, the invader binds to the substrate and displaces the incumbent in a branch migration process. Eventually, the invader completely displaces the incumbent due to the higher thermodynamic stability of the invader–substrate complex. Separating the fluorophore from the quencher leads to an increase in fluorescence intensity, which can be used as a readout of the process. (*b*) TMSD process including a random pool strand that first forms a complex with the invader. The formation of a complex that occludes some of the toehold bases inhibits binding of the invader to the reporter, slowing down the overall displacement kinetics. (*c*) Schematic depiction of TMSD reactions in droplets. Invaders or invader–random pool complexes (green) are co-encapsulated with a reporter complex (orange) inside of a single droplet. After mixing of the droplet content, the TMSD reaction results in an increase in red fluorescence in the droplets. (*d*) Droplet production and monitoring of TMSD. TMSD reactants are encapsulated together in water-in-oil droplets in a microfluidic flow-focusing junction. Droplet sizes and mixing ratios can be controlled via the pressures applied to each inlet reservoir. In order to monitor TMSD reactions within droplets, the droplet flow is stopped instantly by applying a set of balanced pressures between inlets and outlets, directly followed by microscopy data acquisition downstream of the flow-focusing junction.

In the present work, we study micro-compartmentalization [10,11] of a much simpler class of biochemical reactions—toehold-mediated DNA strand displacement (TMSD) processes—which have been extensively studied and used in the context of dynamic DNA nanotechnology over the past two decades [12]. A typical TMSD reaction involves three DNA strands, of which two—the incumbent and the substrate strand—initially are hybridized together in a duplex (cf. figure 1*a*). In TMSD, the incumbent is slightly shorter than the substrate, leaving a short sequence domain of the substrate unpaired (the so-called toehold region). The third strand—the invader—can bind to the toehold and displace the incumbent in a three-way branch migration process. Although simple in concept, TMSD reactions and concatenations thereof have been shown to be remarkably versatile and capable, in principle, of ‘emulating’ the kinetics of any other type of chemical reaction [13]. Among others, TMSD has been used in a wide range of biosensing applications [12], it has been used to drive DNA-based nanomachines and nonlinear reaction dynamics [14], and it has been applied in the context of molecular computing, with implementations ranging from logic gates [15,16] to large neural networks [17]. In applications, TMSD reactions will increasingly have to be operated in complex environments, e.g. as part of larger biochemical networks, as components of protocells [18–21] or even inside cells [22]. In order to assess the impact of a complex reaction environment on TMSD in a comparatively well-defined setting, we recently studied the effect of a pool of DNA molecules with random sequences (as a proxy for a ‘complex environment’) on TMSD reactions. We found that the kinetics of the reactions is significantly affected by the presence of a random sequence background, resulting in a slow-down of the reactions by a factor of over 100 (depending on the concentration of the random pool and other factors [23]). In contrast to the uniform effect exerted by passive molecular crowders such as PEG [24], however, a random pool impacts different sequences differently and will invariably slow down kinetics.

In the present work, we explore the additional effect of compartmentalization into cell-sized droplets on TMSD kinetics in a random pool. While the components of the reaction of interest are present at relatively high concentrations and thus high copy numbers, this is not the case for individual random pool sequences. Each compartment potentially samples a different fraction of the random pool sequence space, which might result in a greater variability of the ‘complex’ compartmentalized system compared with an undisturbed TMSD reaction. In the following, we first investigate the kinetics of a TMSD reaction with and without random pools in a typical bulk experiment. Using droplet microfluidics, we then compartmentalize the TMSD circuit into micrometre-scale droplets of varying sizes. Sampling a large number of droplets allows to accurately assess the mean kinetic behaviour of the compartmentalized circuit as well as its variation between droplets, and thus also investigate the influence of the random pool on the variability.

Figure 1 gives an overview of the basic reactions and the experimental methodology used in this paper. As indicated in figure 1*a*, the kinetics of a TMSD reaction can be monitored by measuring the fluorescence of a fluorophore attached to the substrate, which is initially suppressed by a fluorescence quencher attached to the incumbent. Displacement of the incumbent from the substrate by the invader leads to a separation of fluorophore and quencher and thus an increase in fluorescence. As shown in figure 1*b*, interaction of the invader with a random pool results in the formation of complexes, in which portions of the invader sequence are occluded by a member of the pool. This leads to more complex interactions during the TMSD process and reduced binding of the invader to the toehold, which has been studied in detail in our previous work [23].

For our encapsulation experiments, we used a pressure-controlled microfluidics set-up [25] to encapsulate invader and incumbent–substrate complexes with programmable stoichiometry. Our set-up enables characterization of the droplets using a ‘stopped flow’ approach, which allows fast acquisition of reaction kinetics directly after mixing of the TMSD reaction components (figure 1*c*,*d*). Our experiments show considerable variability in kinetics from droplet to droplet, which can be partially explained by variations in strand stoichiometry that is potentially caused by the production process. However, the variations appear to be considerably enhanced in the presence of a random pool, suggesting an additional influence of the complex reaction environment.

## 2. Methods

### 2.1. Droplet sizes and molecule numbers

In order to be able to observe large variations between different compartments, one would ideally like to perform experiments at very low molecular copy numbers. However, experimentally accessible molecule numbers were constrained by restrictions on the concentration of fluorophores that can be conveniently read out with our fluorescence microscopy set-up, and the size of the droplets that could be reproducibly and stably generated using our microfluidic flow-focusing junction.

The number of molecules *N*_*x*_ of type *x* in a single droplet with radius *r* trivially depends on the droplet volume *V* = (4*π*/3)*r*^3^, and is given by *N*_*x*_ = (4*π*/3) *N*_*A*_ *c*_*x*_ *r*^3^, where *c*_*x*_ is the (molar) concentration of the molecules and *N*_*A*_ is Avogadro’s constant. Our experimental set-up allows monitoring of fluorophores at concentrations in the 100 nM range with acceptable noise levels. For the experiments conducted within this work, we therefore prepared three different stocks, the first containing 400 nM reporter (complexes of incumbent and substrate), the second one containing 500 nM invader (toe 5) and the third containing 500 nM invader (toe 5) and 5 μM random pool strands of 25 nucleotide (nt) length (N25). The random pool sample was pre-incubated for at least 24 h before the experiment for thermodynamic equilibration.

Microfluidic mixing of the stock solutions at different volume ratios—with a fixed final droplet volume—allows one to achieve a range of final concentrations of invaders and reporters. When the stock concentrations of the solutions supplied via the two inlets are [*A*]_stock_ and [*B*]_stock_ and the corresponding volumina merged at the junction are given by *V*_*A*_ and *V*_*B*_, then the constraint *V* = *V*_*A*_ + *V*_*B*_ results in the following concentrations inside the droplets:

$$[A]=\xi {\left[A\right]}_{\mathrm{stock}}$$

and

$$[B]=(1-\xi ){\left[B\right]}_{\mathrm{stock}},$$

where *ξ* := *V*_*A*_/(*V*_*A*_ + *V*_*B*_) is the volume fraction of solution A. The concentrations are thus linked via the relation [*B*] = [*B*]_stock_ · (1 − [*A*]/[*A*]_stock_).

**Figure 2.**
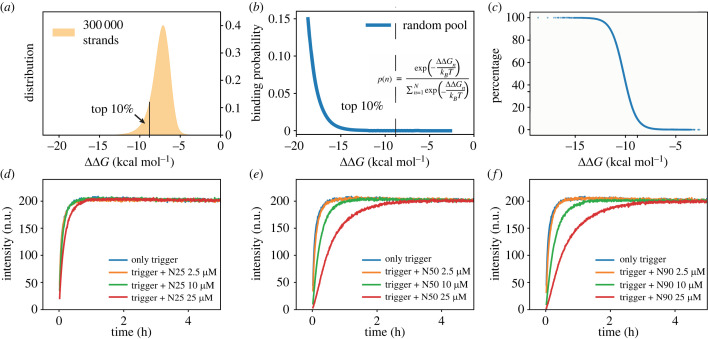
Interactions between invader and random pool strands. (*a*) Distribution of ΔΔ*G* values for the interaction between the invader strand and members of the random pool. NUPACK simulations were performed for 300 000 random pool strands with a length of 25 bases. (*b*) Probability that an invader forms a complex with a random pool strand as a function of the interaction strength. The probability decreases exponentially with ΔΔ*G*. (*c*) Theoretical fraction of invader–random strand complexes in an equilibrated system as a function of the interaction strength. (*d*–*f*) Experimental data for TMSD reactions (as schematically shown in figure 1*b*) for different combinations of pre-equilibrated invader–random pool complexes. Random pool strands of lengths 25, 50 and 90 each with three different concentrations were studied. For the lowest concentration (10-fold excess of RP to trigger), no difference in kinetics is visible, independent of the length of the random pool strands. For the other concentrations, the slowdown is more pronounced for higher concentrations and longer random pool strands (data are given in normalized units (n.u.), normalized to a maximum value of 200).

The total concentration of the random pool was always 10-fold higher than the invader concentration. The radii of the droplets used in this work ranged approximately between *r* = 10 μm and *r* = 30 μm (corresponding to volumes in the range of ≈4–110 pl). Therefore, the number of reporter and invader strands per droplet was in the range from 10^6^ to 10^7^, and the number of random pool strands was accordingly 10-fold higher. As the sequence space of 25 nt long DNA strands is 4^25^ ≈ 10^15^, each droplet can be expected to have a different composition of random strand sequences, and thus only samples a tiny fraction of all possibilities. In contrast to our previous work [23], we chose to only use a tenfold excess of random pool strands, which resulted in only a moderate retardation of the reaction kinetics (see figure 2*d*–*f* for bulk measurements). This allowed us to compare reactions with approximately the same overall velocity and thus ensured that the observed increased variability in the kinetics in the presence of the random pool was not trivially caused by a general slowdown.

### 2.2. Preparation of the TMSD components

The invader strand was ordered PAGE purified and both strands of the reporter were ordered HPLC purified from Integrated DNA Technologies IDT (Coralville, IA, USA). The random pool strands were ordered from IDT (Leuven, Belgium) without purification, choosing machine mixing as the method for the randomization of bases. All experiments were carried out using a 1× TE buffer with additional 100 mM NaCl and 12.5 mM MgCl_2_ (Sigma-Aldrich).


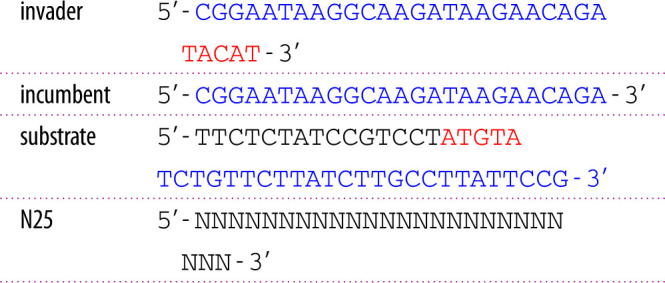


The branch migration domains are depicted in blue and the toeholds in red. The substrate strand is labelled with a 3’ ROX (NHS Ester) fluorophore and the incumbent with a 5’ Iowa BlackRQ quencher. The substrate strand is fully complementary to the incumbent and is extended at the 5’ end by 20 additional unpaired bases, which can act as a toehold for the invader strand. In previous work [23], we had used the same substrate–incumbent complex to investigate TMSD with invaders with different toehold lengths. We found that—in the absence of secondary structure in the toehold—the overall kinetics was only dependent on the number of complementary bases between invader and substrate toehold, and was not affected by the total length of the toehold sequence on the substrate. In the present work, we deliberately only utilized a 5 nt long toehold (marked in red), which slowed down the TMSD process sufficiently to allow fluorescence tracking of the reaction in the droplets. For longer toeholds, the reaction was too fast for reliable data acquisition as within the first few seconds after droplet generation, the droplets are still moving quickly. We also found previously that the interaction of the substrate’s toehold sequence with the random pool only had a comparatively minor effect on the kinetics. As a side note, we comment that with 4^5^ = 1024 the sequence space of the 5 nt toehold is much smaller than for the longer toeholds considered previously. When pool concentrations become excessively high—beyond the level considered in this study—the reaction could be significantly impeded by those members of the pool possessing a subsequence that happens to be complementary to the toehold. For longer toeholds, the entire sequence space cannot be covered by the finite number of molecules in the random pool and therefore its effect will be more nuanced.

### 2.3. Kinetics data treatment

#### 2.3.1. Plate reader data

A BMG FLUOstar Omega plate reader was used to obtain bulk kinetics data from the TMSD systems. All experiments were carried out as technical triplicates to account for experimental variations. The plate reader provides the fluorescence intensity (FI) values in arbitrary units. The FI of the reporter system without any invader was measured to obtain the non-zero background fluorescence value that is caused by imperfect quenching of the fluorophore by the quencher. This value was substracted from each datapoint and the maximum was set to a value of 200 as we used a final reporter concentration of 200 nM and an invader concentration of 250 nM. The final value should thus correspond to the fluorescence of 200 nM unquenched fluorophore.

#### 2.3.2. Microscopy data

In order to obtain kinetic data from fluorescence microscopy measurements, for each droplet the intensity values in the green and red channel were tracked, which corresponded to the fluorescence of a reference fluorophore added to the droplets for normalization (Atto 488, see §2.5) and the fluorophore attached to the substrate strand (ROX). For experiments performed at a constant ratio between invader and reporter strands, we normalized the red channel intensity values with the green channel values for each timestep to account for variations in intensity that are caused by particles moving from one spot to another. This procedure corrects for imperfect flat-field correction and uneven focus across the observation region. For experiments, in which we varied the relative concentrations of the TMSD constituents, normalization using the green reference dye was not feasible as certain droplets contained no or very little amounts of the reference. In these cases, we restricted our analysis to droplets that displayed only very limited motion throughout data acquisition, and for which the illumination thus stayed approximately constant over time.

### 2.4. Preparation of microfluidic devices

Silicon masters were made from 2-inch silicon wafers (Siegert Wafer, Germany) using photo-lithography with an SU8-2025 photoresist (Micro resist Technology) with photomasks (Zitzmann GmbH, 64.000 dpi) designed with AutoCAD. Master wafers were produced in a clean room following manufacturer’s instructions for the desired resist thickness which was measured by a profilometer (Dektak 150 surface profiler, Veeco Instruments). Microfluidic devices were then produced using PDMS soft lithogrpahy. 12 g of PDMS (Sylgard 184, Dow Corning), mixed thoroughly with 1.2 g of its curing agent was poured on a silicon master wrapped in aluminium fold, degassed for 30 min and baked for 1 h at 80°C. Cured PDMS devices were carefully peeled from the master, trimmed with a razor blade and inlet and outlet holes were punched into each device with a biopsy puncher. Glass slides were cleaned thoroughly with 2% Hellmanex III (Hellma) and distilled water then dried for 30 min at 80°C. PDMS devices and glass slides were exposed to O_2_ plasma (1 min, 20 sccm, 100 W) bonded together and baked for 1 h at 80°C.

### 2.5. Generation and monitoring of droplets

All fluorescence microscopy time-lapse data were recorded with a 10X P-Apo air objective (NA 0.45) on a Nikon Ti-2E equipped with a SOLA SM II LED light source, a motorized stage and an Andor NEO 5.5 camera. Temperature was kept constant (*T* = 29°C) in an Okolab microscope enclosure using a H201-T-UNIT-BL temperature controller. All microfluidic channel walls were made hydrophobic by flushing with 1% trichloro(1H,1H,2H,2H-perfluorooctyl)silane (Merck) dissolved in FC-40 oil (Merck) for 1 min immediately prior to use. Microfluidic devices were operated using a 4-channel Elveflow OB1 controller pressurized by a nitrogen gas line (4 bar). Sample reservoirs were connected to the chip with Tygon ND 100-80 tubing (Saint-Gobain, ID 0.5 mm) fitted to the PDMS inlets. The continuous phase contained 2% fluorinated surfactant dissolved in FC-40 oil (FluoSurf, Emulseo). The dispersed phase consisted of two aqueous solutions, containing the reporter complex and the invader strand (pre-incubated or not with random pool strands) supplemented with 1 μM of a fluorescent reference dye (Atto 488, Sigma-Aldrich). The device outlet was also pressure actuated and set to 0 mbar during droplet production. The fluorescence from the reference dye was used for signal normalization, and also to determine the mixing ratio between the invader and reporter solutions, similar in approach as in [25]. When varying the mixing ratio within droplets, symmetric sinusoidal temporal pressure profiles were applied to the aqueous solution reservoirs in order to keep the total pressure constant and thus keep the droplet size unchanged during production, with a typical pressure oscillation period ranging from 0.1 to 5 s. The coefficient of variation of the radii of the droplets varied between 1.3% and 5.9% in our experiments. To monitor the TMSD reactions in the droplets, the field of view was positioned directly downstream of the flow-focusing junction and time-lapse acquisition was started just before applying a set of balanced low pressure values to inlets and outlet (typically, 20 mbar), which stopped the flow of the droplets within a few seconds. Flow resistance sections inserted into the channels for the dispersed phases strongly limited the backflow of oil or aqueous solutions and further helped to reduce fluid motion during acquisition.

### 2.6. Droplet tracking and data analysis

All microscopy images were corrected using background subtraction and flat-field correction in all channels using a custom-made ImageJ macro. Droplets were individually analysed using the segmentation and tracking software TrackMate 7 [26]. Droplets were detected in each frame using an LoG (Laplacian of Gaussian) detector and optimal detection thresholds were determined based on the reference fluorescence signal from the droplets. Droplet sizes were determined using Nikon NIS-Elements software automatic measurement feature. Droplet tracking was performed with a simple LAP tracker and tracking data (spatial coordinates, fluorescence intensity, etc.) were analysed using a custom-made Python script, which removed data that could not be analysed, including droplets with fluorescence traces showing negative slopes, out-of-focus droplets at the upper or lower edges of the field of view or droplets with significant jumps in their intensity profiles.

## 3. Results

### 3.1. Invader–random pool complexes

We numerically studied the properties of complexes containing the invader and one strand of the random pool using the software package NUPACK [27]. In order to generate statistically relevant data, the interaction of *N* = 300 000 randomly generated sequences with a length of 25 nt (N25) with the invader strand was modelled and analysed. Figure 2*a* shows the ΔΔG distribution for the 300 000 random strands. The distribution has a similar shape to that obtained earlier for longer toehold sequences [23], but is slightly shifted towards higher energies. The ΔΔG value measures the binding free energy between invader and each member of the random pool and is given by ΔΔ*G* = Δ*G*_*AB*_ − (Δ*G*_*A*_ + Δ*G*_*B*_), where Δ*G*_*A*,*B*_ are the folding free energies of the isolated strands, and Δ*G*_*AB*_ is the free energy of the complex.

Figure 2*b* shows the probability *p*(*n*) that a specific member R_*n*_ of the random pool with a corresponding ΔΔ*G*_*n*_ will be bound to the invader, which is given by

$$p(n)=Q^{-1}\exp\left(\frac{-\Delta \Delta G_{n}}{k_{B}T}\right),$$

where $Q=\sum_{\;j=1}^{N}\exp(-\Delta \Delta G_{j}/k_{B}T)$. As this probability depends exponentially on the free energy difference, only the random pool strands with the strongest interaction are expected to be in a complex with the invader at thermodynamic equilibrium.

As in this study we applied a 10-fold excess of the random pool strands compared to the invader, we anticipated that only the ≈10% of the random pool population with the lowest ΔΔG would have a major effect. However, it is still possible that not all strands within this 10% subset will form a stable complex with the invader.

In figure 2*c*, we show the fraction *θ*(*C*_*j*_) of invader *A* expected to be in complex with a random strand *R*_*j*_ with corresponding ΔΔ*G*_*j*_, when only considering the binding equilibrium $A+R_{j}⇌C_{j}$. This fraction is directly calculated by NUPACK, but can also be obtained by relating the equilibrium constant to ΔΔ*G*. At the lowest ΔΔ*G* values, almost all of the invaders would be bound in a complex, whereas for higher ΔΔ*G* there is negligible binding. As an estimate for the extent of binding of the invader to the random pool as a whole, we averaged these fractions over the 10% best binders according to $\mu =M^{-1}\sum_{\;j=1}^{M}\theta (C_{j})$, where *M* is the corresponding number of strands, which results in μ≈33%. This suggests that a considerable fraction of the invader will be sequestered by interactions with the random pool. An accurate calculation of the equilibrium concentrations of all possible complexes in the pool is complex, however.

### 3.2. Impact of random pool strands on TMSD

We first studied the impact of random pool strands of different lengths and concentrations on our TMSD reaction in bulk (figure 2*d*–*f*). For the short 5 nt toehold, a significant retardation of the kinetics is only found in the presence of long interfering strands and at large surplus concentrations. For compartmentalization experiments, we therefore decided to use only a 10-fold excess of random pool strands with a length of only 25 nt, which did not noticeably affect the kinetics in the bulk experiment (figure 2*d*).

**Figure 3.**
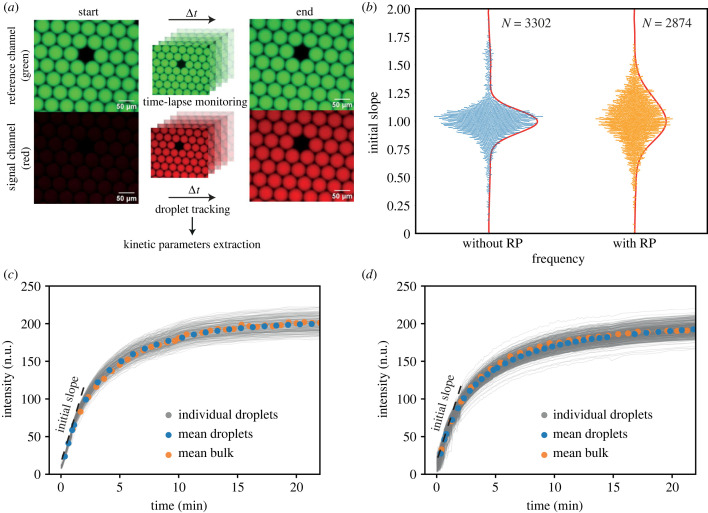
Monitoring TMSD reactions in emulsion droplets. (*a*) Workflow for the extraction of kinetic parameters. Droplets are individually tracked from time-lapse fluorescence microscopy images. Reference (green) and reporter (red) fluorescence intensity values are obtained for each droplet for each time frame. The initial slope of the reporter intensity time course is chosen as a measure for the reaction kinetics. (*b*) Swarm plots of individual slopes after dividing each by the mean value of the set of experiments. The initial slopes show a much larger variability in the presence of a random pool. The red lines indicate Gaussian fits to the data. (*c*,*d*) Kinetic curves of individual TMSD reactions in a set of droplets generated in one experiment. Also shown is the mean of all droplet kinetic curves, and the kinetics for the same TMSD process recorded in a bulk experiment. Experiments were performed without (*c*) and with random pool (*d*), respectively.

Our workflow for the generation of data for micro-compartmentalized TMSD reactions is shown in figure 3*a*. The reaction independent reference dye provides a constant fluorescence signal and can be used for tracking the droplets. The TMSD reaction itself is monitored via the red fluorescence signal, which increases over time due to displacement of the quencher-modified incumbent strand. We obtained several thousand kinetic curves for TMSD reactions with and without random pool strands. Extracting kinetic parameters through curve fitting over the full time course of the TMSD reaction was found unsuitable for fast and efficient data generation, as this would have required monitoring all droplets over a significant timespan—for the TMSD reaction with a 5 nt toehold, ≈45 min are needed to reach a stable fluorescence end-value. During this time, maintaining all droplets immobile and intact within the field of view is challenging due to Ostwald ripening and uncontrolled fluid flows. As our set-up allowed monitoring the droplets immediately after their generation, we decided to assess the speed of the reaction by measuring the initial slope of the kinetic curves as an estimator for the initial reaction velocity *v*_0_ for the majority of our data. The initial velocity *v*_0_ of a second order reaction $A+B\overset{k}{\to }C$ between reactants *A* and *B* is given by the expression *v*_0_ = *k* [*A*]_0_ [*B*]_0_, and is thus connected to the effective kinetic rate *k*.

To improve data quality and robustness, we excluded the first seconds of data acquisition after stopping the droplet flow, which ensured that the droplets were immobile during measurement. For the set of experiments depicted in figure 3, we took the first value (*f*_1_) at *t*_1_ ≈ 10 s and the second value (*f*_2_) after *t*_2_ ≈ 50 s to calculate the initial slope *s* via:

$$s=\frac{\;f_{2}-f_{1}}{t_{2}-t_{1}}\propto v_{0}.$$

We then calculated the mean of all initial slopes, normalized each slope by dividing it by the mean and plotted the result as a swarm plot in figure 3*b*. The experiments with the random pool appear to show a greater variability of initial slopes (and thus reaction kinetics) than the clean system, which suggests that a complex reaction environment might result in an increased diversity among the compartmentalized reactions.

Figure 3*c*,*d* compares the kinetic curves obtained in individual experiments with and without the random pool and their corresponding mean curves with the kinetics obtained from platereader experiments containing the same reaction compositions. Satisfyingly, the mean kinetics obtained from the droplet experiments coincide very well with the bulk kinetics, validating our general experimental approach. The second-order kinetic rates derived from the data were found to be *k* = 3.2 × 10^4^ M^−1^ s^−1^ and *k* = 2.7 × 10^4^ M^−1^ s^−1^ in the absence and presence of the random pool, respectively, which is in good agreement with previous work on TMSD processes with 5 nt long toeholds. The random pool effectively slows down the TMSD reaction by ≈20%.

Individual experiments display considerable, symmetric scatter around the mean behaviour, which in agreement with the data in figure 3*b* appears to be larger for the random pool case. To quantify the variations, we calculated statistical measures such as the coefficient of variation, defined as the standard deviation divided by the mean (CV_*s*_ = Δ*s*/〈*s*〉), or the interquartile range (IQR), which is the difference between the 75th and 25th percentiles of the data. Numerically, the random pool sample only shows a slightly enhanced CV compared to the clean sample (CV = 0.247 versus CV = 0.226, respectively), but they differ notably in IQR (IQR = 0.258 versus IQR = 0.163) or the mean absolute deviation (0.00025 versus 0.00017). The relatively large CV of the clean sample is caused by outliers with s≳1.5 (cf. Figure 3*b*). If instead we only consider the central peak of the distribution (indicated by Gaussian fits in the figure), we obtain a much smaller CV ≈ 0.1 in the absence of random strands as opposed to CV ≈ 0.15 in their presence. Simple error propagation of the expression for the initial velocity shows that the CV = Δ*s*/*s* = Δ*v*_0_/*v*_0_ is affected by variations of the rate constant and the strand concentrations:

$$\frac{\Delta v_{0}}{v_{0}}\approx \frac{\Delta k}{k_{0}}+\frac{\Delta {\left[A\right]}_{0}}{{\left[A\right]}_{0}}+\frac{\Delta {\left[B\right]}_{0}}{{\left[B\right]}_{0}}.$$

As Δ[*A*]_0_ = Δ[*B*]_0_ is expected to be the same for both random pool and clean samples, we attribute the enhanced droplet-to-droplet variability observed in the random pool droplets to differences in the (effective) rate constant. We hypothesize that the additional variability is caused by partitioning effects that results in an undersampling of the random sequences. Each droplet samples a different selection of complexes between TMSD and random pool strands, which also vary in their sequence and therefore interaction properties, which in turn affects the kinetics of the TMSD reaction.

### 3.3. Kinetic mapping of TMSD reaction

We further hypothesized that pressure fluctuations during the droplet generation process would result in variable droplet compositions. We therefore decided to systematically study TMSD reaction rates for different ratios between invader strands (with added random pools) and reporter complexes. To this end, we dynamically varied the mixing ratios in the droplets by applying opposing sinusoidal pressure profiles to the two inlets for the aqueous solutions.

**Figure 4.**
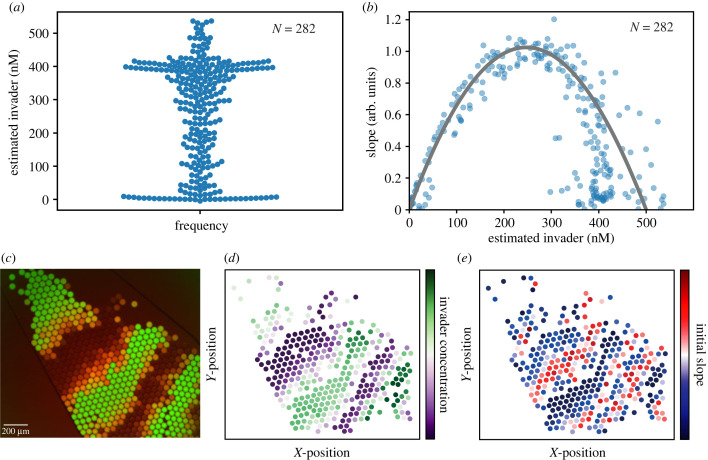
(*a*) Swarm plot of estimated invader concentration for the collection of droplets shown in (*c*). (*b*) Scatter plot of the initial slopes for each droplet versus the corresponding estimated invader concentration (blue dots). The grey parabola is the theoretically expected dependence of the slope on the invader concentration (see text). (*c*) Composite fluorescence microscopy image of halted droplets produced with different mixing ratios around 100 s after generation (green: reference dye; red: reporter dye). (*d*) Spatial map of the estimated invader concentration in the tracked droplets of the left panel. The concentrations follow the sinusoidal pattern imposed by the control pressure protocol. (*e*) Spatial map of the initial slope of the tracked droplets. The slope is highest in regions with approximate stoichiometry between invader and reporter.

We estimated the actual invader concentration in each droplet by using the mean of the 10 lowest reference dye fluorescence intensities as the value for zero invader concentration, and the mean of the ten highest intensities as the maximum concentration values, and linear interpolation of the intermediate concentrations (figure 4*a*). The fluorescence intensity of the Atto 488 reference dye is proportional to the volume of invader stock solution added to a droplet and thus provides an estimate of the invader concentration that is not influenced by the reaction inside the droplet itself.

We then determined the initial slopes for a wide range of invader/random pool and reporter ratios. For this set of experiments, we derived the slope from the larger time interval Δ*t* ≈ 10−90 s as fluctuations were common for traces with large imbalances between invader and reporter concentrations.

The measured slopes as a function of the invader strand concentration nicely follow a parabola, which is actually expected for a second order reaction with varying ratio between the reactants. Using the expressions for the droplet concentrations derived above, this translates into *v* = *kξ*(1 − *ξ*)[*A*]_stock_ [*B*]_stock_, which is a quadratic function of the volume ratio *ξ* = [*A*]/[*A*]_stock_. The velocity is thus expected to be maximal for a volume ratio of *ξ* = 1/2, or [*A*] = [*A*]_stock_/2.

In order to more closely model our experimental approach, in which we determined the initial slope of the reaction kinetics within a finite time interval, we numerically solved the corresponding ordinary differential equations describing the TMSD process, i.e.

$$\dot{\left[I\right]}=-k[I][R]=\dot{\left[R\right]}=-\dot{\left[F\right]},$$

where [*I*] denotes the concentration of the invader complexes (including free invaders and invaders bound to random pool strands), [*R*] is the concentration of the reporter complex, and [*F*] denotes the concentration of the fluorescent invader–substrate complex. For the simulation, we used the second order rate constant *k* determined from the fit to the mean curve of the experiment depicted in figure 3*d*. We solved the equations for varying invader and reporter concentrations using a Python-integrated ODE solver and extracted the slopes for the same Δ*t* as in the experiments. The values for the slopes follow the grey parabola shown in figure 4*b*. In order to match the curve with the experimental data, it was scaled by a factor accounting for the conversion of the measured fluorescence intensities to the molar concentrations of the simulation, but otherwise no curve fitting was involved. As expected, the parabola has its maximum for a volume ratio of *ξ* = 1/2, corresponding to an invader concentration of [*I*] = 250 nM. When applying pressure oscillations at a sufficiently high frequency with respect to the droplet generation rate, droplets with the whole range of TMSD component ratios can be captured in a single field of view (figure 4*c*). Spatial maps of the droplet composition and the corresponding kinetic slopes visualize that the fastest kinetics are observed in droplets with an intermediate invader concentration.

The experiments demonstrate that experimental factors such as pressure variations in the fluidic channels can affect droplet composition, which in turn results in variations in the observed TMSD kinetics. We assume that these variations are a major contribution to the variability observed in figure 3*b*. However, the presence of the random pool appears to also affect the variability and results in a broadened distribution of the reaction velocities inside the droplets.

## 4. Conclusion

In this work, we have studied the kinetics of toehold-mediated strand displacement reactions encapsulated in emulsion droplets, in both the absence and presence of interfering DNA strands taken from a random sequence pool, using a dedicated microfluidic ‘stopped flow’ set-up. One of the major advantages of this approach is the observation of a large number of replicas of the same reaction in a single experiment, and the possibility to screen experimental parameters—such as the stoichiometry among the encapsulated components—very quickly.

Investigating the kinetics in thousands of droplets shows considerable variability among the droplets, which can be explained, in part, by variations in the stoichiometry between the TMSD components, which might be caused by the microfluidic droplet production process. Notably, the presence of a random sequence pool co-encapsulated with the TMSD components seems to also affect the reaction kinetics in the droplets, which is particularly remarkable as the molecule numbers of the TMSD components themselves are orders of magnitude too high to result in notable stochastic reaction dynamics. We speculate that each of the droplets effectively samples a slightly different subset of the random pool, causing an increased variability in reaction kinetics. In the present work, we only added the random pool in tenfold excess, and we used 5 nt long toeholds, which allowed us to observe the kinetics on an experimentally accessible timescale. Previous work with longer toeholds and higher random pool concentrations [23] showed a much stronger impact on kinetics, and this might also lead to a more pronounced variability when compartmentalizing such reactions.

On a more abstract level, the random sequence pool represents a large collection of molecules that interferes with a molecular circuit of interest via transient binding with a wide distribution of different interaction strengths. It might therefore also be taken as a proxy for a complex biochemical environment in general, which will inevitably be involved in any attempt to create cells from the bottom up. To effectively deal with such effects in synthetic cells, strategies to control, circumvent or even use such effects will need to be developed.

## Data Availability

Data and scripts are available on request. The Python code used for the analysis of the experiments has been deposited in the Github repository: https://github.com/TomTom9595/Royal-Society-Interfaces.
